# Function of snail shell hairs in anti-predator defense

**DOI:** 10.1007/s00114-024-01901-z

**Published:** 2024-02-27

**Authors:** Nozomu Sato, Akihiro Yoshikawa

**Affiliations:** 1 Graduate School of Urban Environmental Sciences, Tokyo Metropolitan University, 1-1 Minami-osawa, Hachioji city, Tokyo, 192-0397 Japan; 2 Amami-city, Kagoshima Japan

**Keywords:** Firefly, Hairy snail, Hair-like structure, Periostracum, Predator–prey interaction, Pygopodium

## Abstract

The function and evolutionary background of the hairs on the shells of terrestrial gastropods is largely unknown. Many hypotheses proposed by malacologists have never been proven, and the long-held hypothesis of mechanical stability in wet environments has been rejected by recent studies. It would therefore be worthwhile to reexamine other hypotheses regarding the adaptive significance of shell hairs. We investigated the defense function of shell hairs against a specialist predator, the snail-eating firefly, in the long-haired snail *Moellendorffia diminuta*. The firefly larvae, which hunt snails using abdominal suckers, were unable to attach to the shell because of the shell hairs but were able to attach to the shells that had lost their hairs. About half of the hairy snails successfully defended themselves by swinging their shells and dropping firefly larvae, but most of the snails without hair failed to defend. The hairs reduce the ability of the larva to attach to the shell and increase the effectiveness of the shell-swinging defense behavior in removing the larva from the shell. As shell hairs grow longer with shell development, they may confer an advantage based on the predator’s growth stage. Our findings highlight the anti-predator defense role of shell hairs in land snails, introducing a hypothesis previously overlooked in the evolutionary context of hairy snails.

**Supplementary Information:**

The online version contains supplementary material available at 10.1007/s00114-024-01901-z.

## Introduction

Animal hairs and hair-like structures serve as defenses, coordinate movement, and function as a diverse array of sensors (Emlen 2014; Sugiura and Yamazaki 2014; Boublil et al. 2021). However, the function of “hairs” on the shells of terrestrial gastropods remains poorly understood. Shell hairs are semi-rigid structures that are part of the periostracum covering the calcareous shell, and this high energy cost-producing structure has evolved independently in several families of land snails (Pfenninger et al. 2005). Malacologists have proposed various hypotheses regarding the adaptive significance of hairs in hairy snails (Schilthuizen 2003; Pfenninger et al. 2005; Dourson 2013), but empirical studies on these hypotheses are lacking. Until recently, shell hairs were thought to have evolved by their selective advantage in providing mechanical stability on wet plants (Pfenninger et al. 2005). However, the thorough examination of Shvydka et al. (2020) revealed limited connections between hairs and substrate adhesion, challenging this notion. Therefore, alternative hypotheses regarding the function of shell hairs merit further testing. Shell modifications found in a few land snails are known to be protective against predators that burrow into the shell (Liew and Schilthuizen 2014), while the protective effect of soft hairs is underestimated compared to these harder structures.

**Fig. 1:**
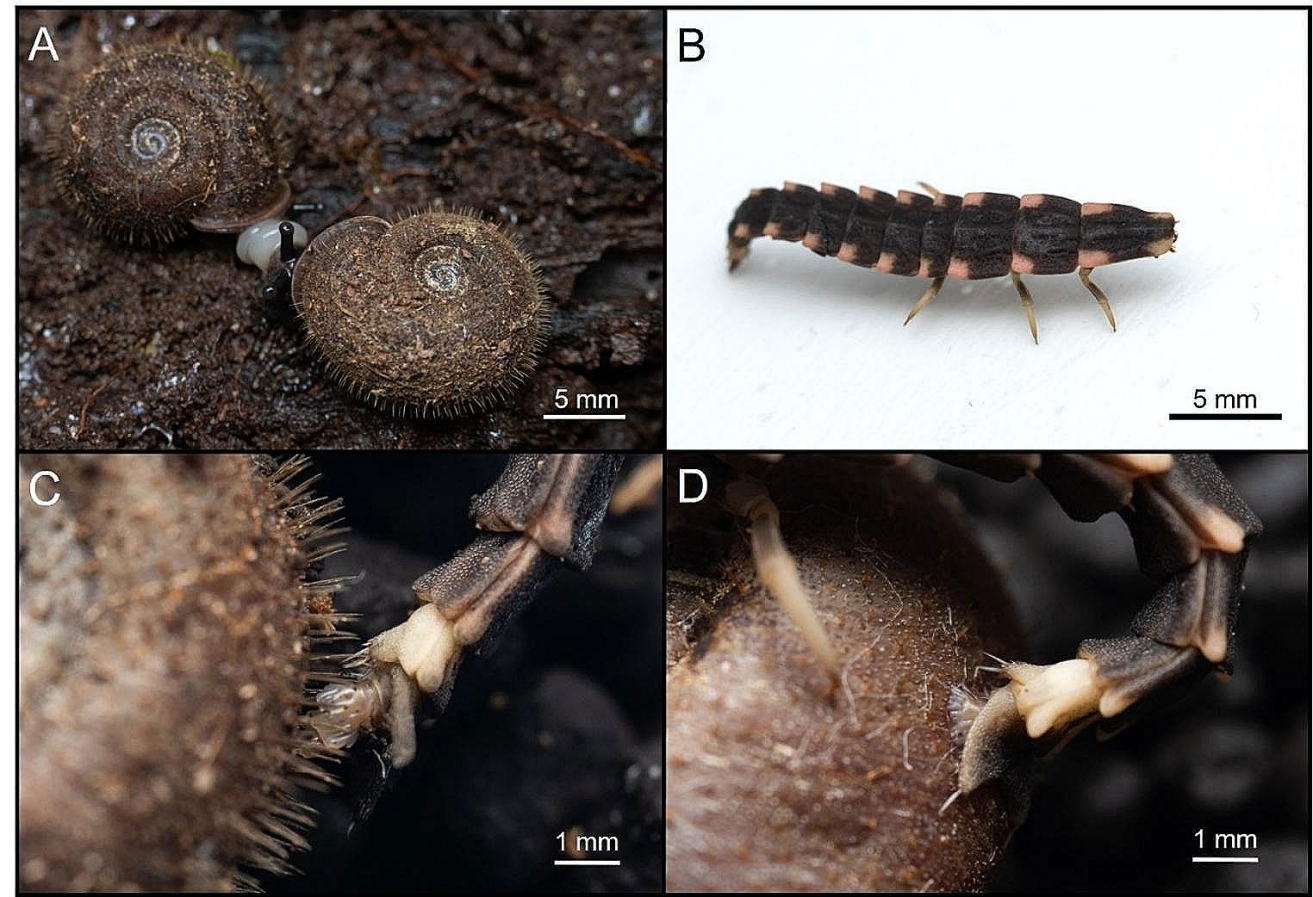
Hairy snail and snail-eating firefly. (**A**) *Moellendorffia diminuta.* (**B**) *Pyrocoelia oshimana* larva. (**C**) Shell hairs prevent attachment of the larval pygopodium. (**D**) Larval pygopodium attached to a hairless shell

Here we focused on the snail-eating firefly, a shell-riding predator, to test the anti-predator defense function of shell hairs. On Amami-Oshima Island in Japan, *Pyrocoelia oshimana* (Coleoptera: Lampyridae) is widespread on the subtropical forest floor and preys on the sympatric long-haired endemic land snail *Moellendorffia diminuta* (Stylommatophora: Camaenidae) (Fig. 1A, B). Firefly larvae search for prey by tracking a mucus trail and hunt land snails by riding on the smooth shell with their sucker-like organ, the “pygopodium,” at the abdominal end (Wang et al. 2007; Sato 2019; Riley et al. 2021). The genus *Pyrocoelia*, after fixing its pygopodium securely on the shell, bites the soft body parts of the land snail and injects venom and digestive juices, gradually weakening its prey. In response, the land snail resists by shaking its shell and trying to drop the firefly larva (Sato 2019). Therefore, these shell hairs are expected to impede the attachment of the larval pygopodium to the shell and contribute to their escape from predation.

## Materials and methods

Between June 2021 and December 2022, we collected *M. diminuta* and *P. oshimana* from subtropical forests below 200 m elevation on the north side of Amami–Oshima Island for a predation experiment (Online Resource l, Fig. S1). Samples were also collected in June 2023 for larval pygopodium measurements. To compare predator’s morphology between islands, larvae of *Pyrocoelia matsumurai matsumurai* were also collected in the southern part of Okinawa Island (without long-haired snails like *Moellendorffia*) in October 2023.

To examine the defense function of shell hairs, predation experiments were conducted using a group with clipped hairs (shell diameter: 12.6 ± 2.1 mm; shell height: 6.9 ± 0.7 mm; weight: 0.51 ± 0.18 g; *N* = 28) and a control group with intact hairs (12.4 ± 2.7 mm; 6.7 ± 0.8 mm; 0.47 ± 0.20 g; *N* = 23). To prevent periostracum damage, only the shell hairs were carefully cut using an electric face shaver for downy hairs. The experiment was performed 2 days after this hair treatment. *P. oshimana* larvae were used for both the group without hairs (body length: 20.2 ± 4.6 mm; weight: 0.066 ± 0.39 g; *N* = 28) and with hairs (body length: 19.4 ± 4.5 mm; weight: 0.065 ± 0.042 g; *N* = 23). Experimental design follows Sato (2019), one snail and one larva were placed in a sealed experimental container (10 cm in diameter and 4 cm in height), with behavior recorded overnight (12 h) using an infrared camera under dark room conditions. Defense success or failure against the initial attack of the larvae was recorded, with “Defense success” recorded if the snail shook off the larvae and escaped, and “Defense failure” recorded if the snail was trapped in the shell and remained motionless for more than 30 min, indicating predation. Trials with no larval attack were excluded from the analysis. Surviving individuals persisted for at least 2 weeks after the experiment.

To determine the relationship between the shell hairs of *M. diminuta* and the pygopodium of *P. oshimana*, the lengths of these traits were measured. In addition, we compared pygopodium morphology among two closely related *Pyrocoelia* species (Osozawa et al. 2015) on islands with and without *Moellendorffia* to detect if there is predator adaptation for long shell hairs. Given that shell hairs grow with shell development, the mean length of 10 hairs in the body whorl near the shell aperture of *M. diminuta* (shell diameter range: 5.8–15.4 mm, *N* = 40) was measured. The pygopodia of *P. oshimana* (3rd–6th instar, *N* = 17) and *P. m. matsumurai* (3rd–5th instar, *N* = 15) were fixed by placing the larva between the cover glass and glass slide, observing from the ventral side. Pygopodia are membranous tubules outlined by rows of microscopic hooks, called multitubular holdfast organs, and are found in all lampyrid larvae (Lawrence et al. 2011; Riley et al. 2021). The mean length of five branchial pygopodium was measured, with these measurements performed on different individuals than those used in the predation experiment owing to the stressful nature of the procedure.

Statistical analyses were performed using R 4.3.0 (R Development Core Team 2023). Fisher’s exact test was used to determine significant differences in defense success rates between snails with and without hairs. Analysis of covariance (ANCOVA) was used to examine differences between the slopes and intercepts of two regressions for the mean length of pygopodium and body length of *P. oshimana* and *P. m. matsumurai*.

## Results

**Fig. 2:**
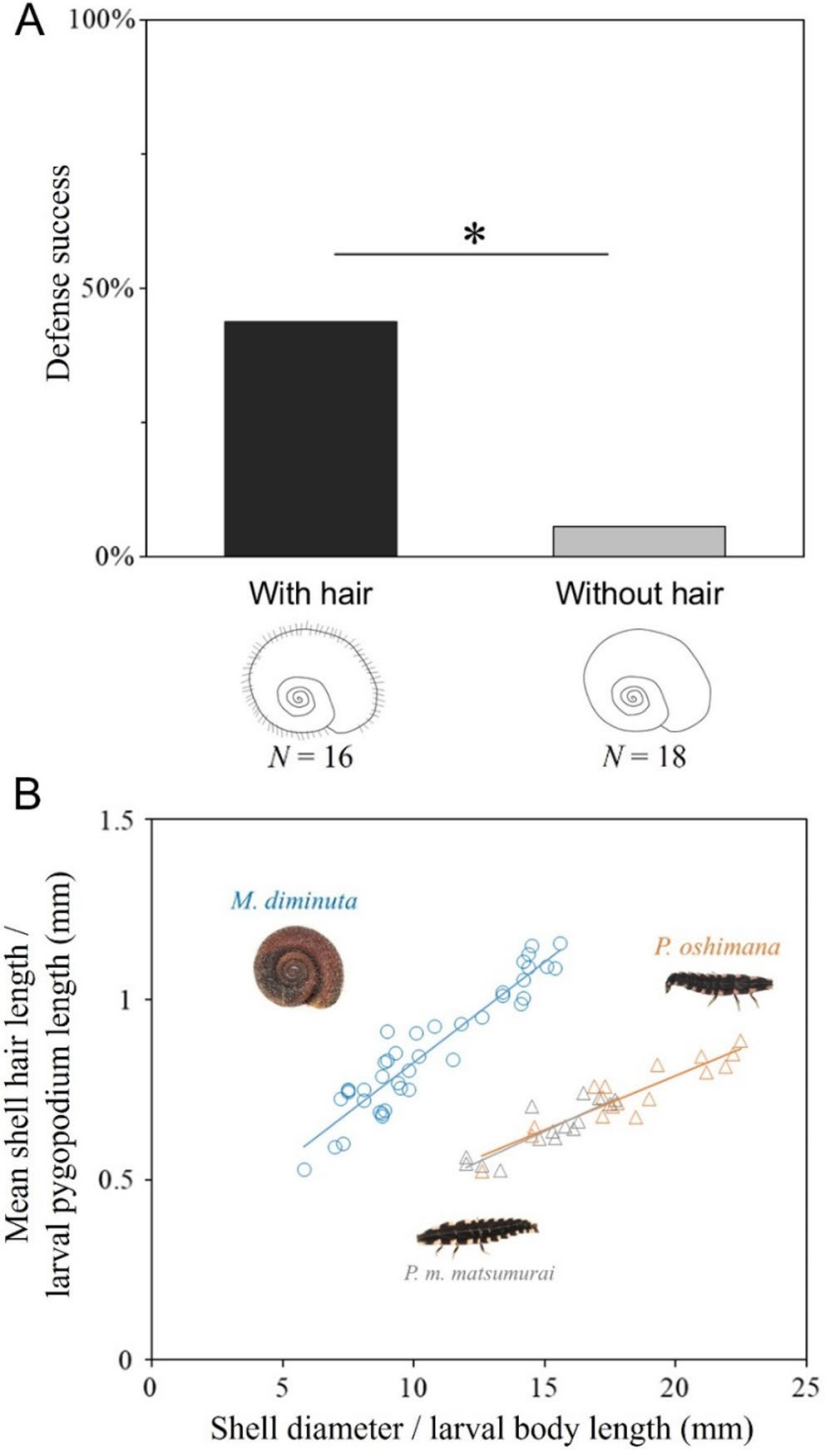
Successful defense against firefly larvae attack in hairy snails and the relationships between shell hair and pygopodium length. (**A**) Success rate of defense in *M. diminuta* with and without hairs. “*” indicates significant difference (*p* < 0.05) based on Fisher’s exact test. (**B**) Relationship between shell hair length and the mean pygopodium length of *Pyrocoelia* larvae. Approximate linear relationships: *M. diminuta* (*y* = 0.0559*x* + 0.2678, *r*^*2*^ = 0.8854, *p* < 0.001, *N* = 40), *P. oshimana* (*y* = 0.03*x* + 0.1876, *r*^*2*^ = 0.8433, *p* < 0.001, *N* = 17), and *P. matsumurai matsumurai* (*y* = 0.0334*x* + 0.1341, *r*^*2*^ = 0.7632, *p* < 0.001, *N* = 15)

In laboratory experiment, *M. diminuta* exhibited a shell-swinging defense behavior when firefly larvae mounted and attacked the snail. This defense effectively dislodged the larvae, enabling escape from predation on multiple occasions. There were trials with no larval attacks during the experiment, 7 cases in snail with hair and 10 cases in snail without hair (Online Resource l, Table S1). Snails with hairs blocked larval pygopodium attachment (Fig. 1C), whereas the pygopodium remained attached to the shell in snails lacking hairs (Fig. 1D). Snails with hairs achieved a successful defense rate of 43.75%, whereas those without hairs struggled to dislodge larvae by swinging their shells, resulting in a low successful defense rate of 5.56% (Fisher’s exact test: *p* = 0.0145) (Fig. 2A).

Snail shell hairs lengthen with shell growth, exceeding 1 mm in adult snails (Fig. 2B; Online Resource l, Tables S2 and S3). Conversely, the pygopodium of *P. oshimana* larvae allometrically lengthens with larval size but remains shorter than the adult snail hairs, even in final instar larvae. The pygopodium of *P. m. matsumurai*, a closely related species on Okinawa Island without *Moellendorffia*, shows a comparable size to that of *P. oshimana*, with no difference in the slope and intercept of the regression relationship between the two species (Analysis of covariance: slope: *df* = 1, *F* = 0.2956, *p* = 0.591; intercept: *df* = 1, *F* = 8e − 4, *p* = 0.9775).

## Discussion

Our observations indicate that the hair-like structure of snail shells prevents predators, such as firefly larvae, from attaching to the shell, thereby enhancing the effectiveness of shell-swinging defense as an anti-predator behavior. Some snails exhibit defensive behavior against firefly larvae by swinging their shells vigorously from side to side, making contact with the surrounding substrate (Wang et al. 2007; Sato 2019). However, as this study was conducted in a flat experimental arena with no obstacles, the effectiveness of the shell-swinging defense may have been underestimated. Notably, *M. diminuta* is active under fallen trees and in crevices, where the complex substrate environment likely amplifies the effectiveness of this defense.

The shell hairs of *M. diminuta* are long enough to prevent the attachment of pygopodium of the *Pyrocoelia* larvae and may accommodate the different growth stages of the predator. On the other hand, there was no difference in pygopodium size between *P. m. matsumurai* and *P. oshimana*, indicating no strong selection pressure for this trait. This may be because firefly larvae prey on a variety of land snail species, and generally favoring prey smaller than their own body size (Fu and Meyer-Rochow 2013; Sato 2019). Pygopodium size may not be decisive in predation of smaller hairy snails, as large larvae can capture small shells by cradling them with their feet without pygopodium attachment. Moreover, hairs in hairy snails are well-developed in juveniles but wear off in old age (Pfenninger et al. 2005; Cameron 2016). Shell hairs associated with shell and periostracum growth are not regenerated once worn away, so older individuals and snails that have lost their hairs may be more vulnerable to predators. This study suggests that shell hairs are advantageous when size differences with fireflies are not substantial, increasing the likelihood of survival or escape from predators attached to the shell.

Importantly, our results support the anti-predator defense hypothesis of shell hairs, especially in species with dense and long hairs like *M. diminuta*; therefore, do not explain hair function in other broadly defined hairy snails (e.g. fine or partial hairs). Many hairy snails inhabit highly acidic tropical forests that cause calcium dissolution in their shells, and fine hairs and thick periostraca may help protect shells (Bullis et al. 2020; Bichain et al. 2022). However, this does not clarify why other land snails lack hairs. Considering the abundance of snail-eating predators in moist forest environments where hairy snails are found, shell hair length may be related to the traits of these predators and the intensity of interspecific interactions. Further evidence is needed to elucidate why shell hairs evolved in hairy snails, considering both environmental factors and the history of biological interactions.

### Electronic supplementary material

Below is the link to the electronic supplementary material.

Supplementary Material 1

## Data Availability

All data generated or analyzed during this study are included in this published article and supplementary information files.
