# Supplementary material for: Function of snail shell hairs in anti-predator defense
Source: Naturwissenschaften. 2024 Feb 27;111(2):13. doi: 10.1007/s00114-024-01901-z (PMC10899286; doi:10.1007/s00114-024-01901-z)
Supplement: Supplementary file 1 — Supplementary Material 1 [file 114_2024_1901_MOESM1_ESM.pdf]

Function of snail shell hairs in anti-predator defense

The Science of Nature, Naturwissenschaften

Authors: Nozomu Sato*[1], Akihiro Yoshikawa[2]

Affiliations:
[1]Graduate School of Urban Environmental Sciences, Tokyo Metropolitan University, 1-1 Minami-osawa, Hachioji city, Tokyo 192-0397, Japan
[2]Amami city, Kagoshima, Japan

*E-mail address: luciolalights@gmail.com

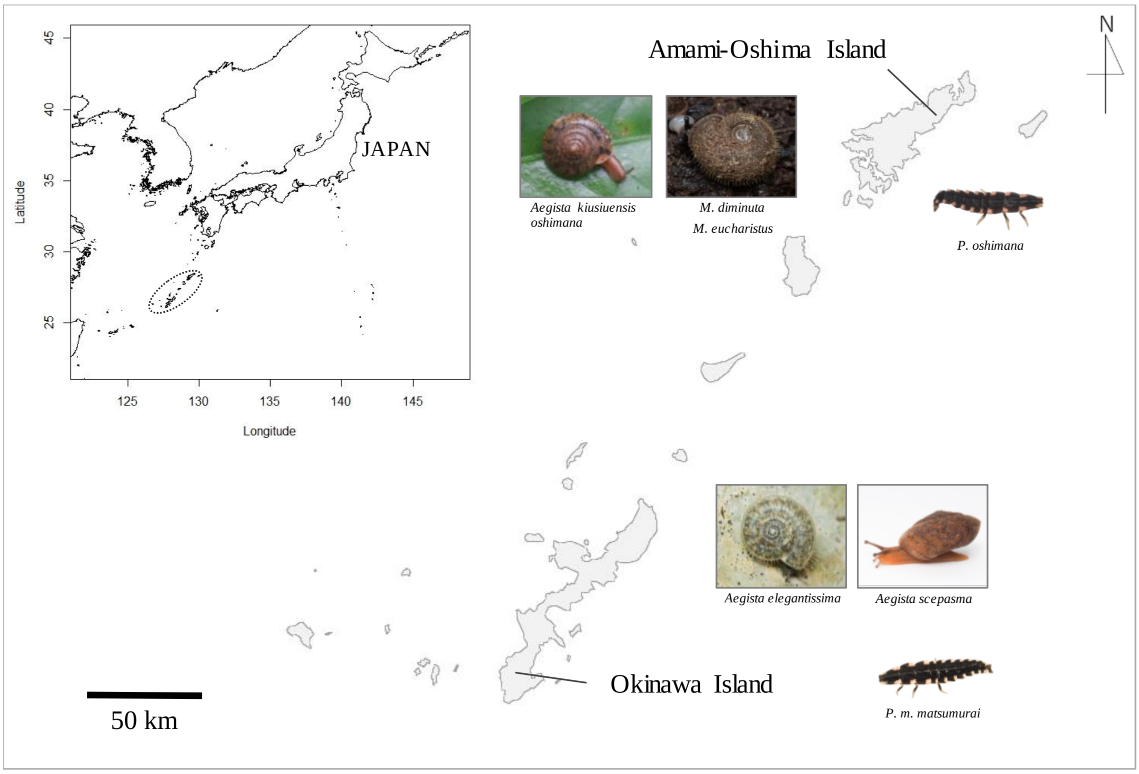

Fig. S1 Survey area.

Amami-Oshima Island (28°19'35"N, 129°22'28"E) and Okinawa Island (26°28′46″N, 127°55′40″E) are the inhabited islands that form southwestern archipelago of Japan. At least three hairy snails are present on Amami-Oshima Island. In contrast to the long-haired *Moellendorffia* species, *Aegista kiusiuensis oshimana* has only short hairs on the margins of its shell. Many hairy snails in the genus *Aegista* are found on the Okinawa Island (Hirano et al. 2014; Okinawa Prefecture 2017). For example, *A. elegantissima* having long hairs only on the margins and *A. scepasma* having short scale-like hairs on the entire shell. Several species of the genus *Japonia* (Architaenioglossa: Cyclophoridae) with rows of setae are also known. However, there are no species with dense long hairs on the entire shell like *Moellendorffia*. Each island has a closely related endemic *Pyrocoelia* firefly (Osozawa 2015).

Table S1 Data for predation experiment.

	Moellendorffia diminuta				*Pyrocoelia oshimana* larva		Defense success
No.	Shell hairs	Shell diameter (mm)	Shell height (mm)	Weight (g)	Body length (mm)	Weight (g)	
1	With	14.1	6.9	0.440	26.2	0.135	F
2	With	15.4	7.8	0.568	26.9	0.146	F
3	With	15.2	7.3	0.594	24.2	0.131	-
4	With	13.8	7.5	0.551	17.9	0.063	S
5	With	8.3	5.5	0.162	13.9	0.014	S
6	With	9.7	6.2	0.219	14.3	0.019	F
1	With	15.1	7.2	0.661	23.6	0.071	-
2	With	15.6	7.7	0.822	24.4	0.111	-
3	With	9.0	5.7	0.248	18.2	0.043	-
7	With	8.3	5.5	0.162	13.9	0.014	F
8	With	14.2	7.1	0.643	22.4	0.074	F
9	With	15.4	8.1	0.682	24.1	0.112	S
10	With	10.5	6.2	0.307	14.9	0.023	S
11	With	8.1	5.2	0.192	14.1	0.014	S
12	With	13.1	6.9	0.598	21.6	0.056	F
13	With	9.2	5.4	0.233	14.4	0.017	-
14	With	9.5	6.1	0.254	12.0	0.017	S
15	With	13.5	6.9	0.632	23.9	0.096	F
16	With	13.4	7.4	0.574	20.4	0.084	S
17	With	10.2	6.5	0.326	14.3	0.020	-
18	With	14.1	7.4	0.649	19.5	0.059	F
19	With	14.6	7.6	0.688	21.1	0.087	-
20	With	13.9	7.0	0.626	20.5	0.088	F
21	Without	13.6	6.8	0.491	22.0	0.091	-
22	Without	9.2	5.7	0.278	14.0	0.019	F
23	Without	14.0	7.4	0.634	22.1	0.079	F
24	Without	14.0	7.2	0.508	27.6	0.132	-
25	Without	13.9	7.3	0.654	22.5	0.081	F
26	Without	14.5	7.6	0.694	24.6	0.149	F
27	Without	13.9	7.6	0.631	25.2	0.109	F
28	Without	10.7	6.1	0.310	13.6	0.019	-
29	Without	10.3	6.4	0.283	14.5	0.024	-
30	Without	8.4	5.4	0.149	14.6	0.014	-
31	Without	14.0	7.1	0.536	26.5	0.120	F
32	Without	13.5	7.3	0.567	26.2	0.095	-
33	Without	10.4	6.2	0.292	13.7	0.019	-
34	Without	14.6	7.6	0.656	23.2	0.078	F
35	Without	13.7	7.5	0.444	24.2	0.109	F
36	Without	9.1	5.4	0.200	14.2	0.022	-
37	Without	13.7	7.5	0.693	21.0	0.063	F
38	Without	14.5	7.9	0.694	24.2	0.078	F
39	Without	10.6	6.5	0.373	16.8	0.041	F
40	Without	14.6	7.3	0.738	24.9	0.108	F
41	Without	15.2	7.8	0.781	23.0	0.095	F
42	Without	14.4	7.7	0.759	21.3	0.082	F
43	Without	11.6	6.4	0.423	16.5	0.030	-
44	Without	10.2	6.3	0.359	14.1	0.032	-
45	Without	9.9	6.4	0.314	14.0	0.018	F
46	Without	14.1	6.6	0.588	21.7	0.081	F
47	Without	13.9	6.9	0.682	20.3	0.031	S
48	Without	11.3	6.6	0.457	18.9	0.039	F

Snail defense results are shown as defense success(S), failure (F), and no larval attack (-).

Table S2 Raw data for *Moellendorffia diminuta.*

No.	Species	Shell diameter (mm)	Mean hair length (mm)
1	*M. diminuta*	14.5	1.149
2	*M. diminuta*	14.4	1.127
3	*M. diminuta*	13.4	1.019
4	*M. diminuta*	15.4	1.086
5	*M. diminuta*	14.2	1.055
6	*M. diminuta*	14.2	1.004
7	*M. diminuta*	14.2	1.106
8	*M. diminuta*	10.1	0.907
9	*M. diminuta*	10.2	0.842
10	*M. diminuta*	8.9	0.827
11	*M. diminuta*	9.5	0.754
12	*M. diminuta*	9.8	0.803
13	*M. diminuta*	8.1	0.751
14	*M. diminuta*	11.5	0.832
15	*M. diminuta*	11.8	0.931
16	*M. diminuta*	9.3	0.852
17	*M. diminuta*	8.9	0.691
18	*M. diminuta*	7.2	0.725
19	*M. diminuta*	8.1	0.718
20	*M. diminuta*	7.3	0.599
21	*M. diminuta*	7.0	0.590
22	*M. diminuta*	8.8	0.786
23	*M. diminuta*	8.7	0.686
24	*M. diminuta*	7.5	0.748
25	*M. diminuta*	8.8	0.683
26	*M. diminuta*	8.8	0.675
27	*M. diminuta*	9.8	0.750
28	*M. diminuta*	7.5	0.750
29	*M. diminuta*	5.8	0.528
30	*M. diminuta*	7.5	0.742
31	*M. diminuta*	9.0	0.830
32	*M. diminuta*	9.4	0.769
33	*M. diminuta*	12.6	0.950
34	*M. diminuta*	13.4	1.010
35	*M. diminuta*	10.8	0.926
36	*M. diminuta*	14.4	1.089
37	*M. diminuta*	15.1	1.090
38	*M. diminuta*	15.6	1.155
39	*M. diminuta*	9.0	0.911
40	*M. diminuta*	14.1	0.987

Table S3. Raw data for *Pyrocoelia* fireflies.

No.	Species	Body length (mm)	Mean pyopodium length (mm)
1	*P. oshimana*	21.2	0.797
2	*P. oshimana*	17.8	0.713
3	*P. oshimana*	17.6	0.703
4	*P. oshimana*	17.3	0.759
5	*P. oshimana*	22.5	0.885
6	*P. oshimana*	16.9	0.758
7	*P. oshimana*	21.0	0.841
8	*P. oshimana*	21.9	0.814
9	*P. oshimana*	19.0	0.724
10	*P. oshimana*	19.3	0.818
11	*P. oshimana*	22.2	0.849
12	*P. oshimana*	18.5	0.674
13	*P. oshimana*	17.2	0.677
14	*P. oshimana*	12.6	0.524
15	*P. oshimana*	14.5	0.623
16	*P. oshimana*	14.6	0.645
17	*P. oshimana*	17.2	0.728
1	*P. m. matsumurai*	16.5	0.739
2	*P. m. matsumurai*	17.5	0.711
3	*P. m. matsumurai*	16.3	0.662
4	*P. m. matsumurai*	17.1	0.727
5	*P. m. matsumurai*	12.6	0.538
6	*P. m. matsumurai*	14.8	0.613
7	*P. m. matsumurai*	15.8	0.647
8	*P. m. matsumurai*	15.3	0.634
9	*P. m. matsumurai*	13.3	0.525
10	*P. m. matsumurai*	17.7	0.723
11	*P. m. matsumurai*	14.5	0.703
12	*P. m. matsumurai*	16.1	0.641
13	*P. m. matsumurai*	12.0	0.562
14	*P. m. matsumurai*	12.0	0.545
15	*P. m. matsumurai*	15.4	0.614

Ethics

The experiments were undertaken according to the animal ethics of Japan and Tokyo Metropolitan University. Samples were collected outside the protected area because Amami–Oshima Island and Okinawa Island are part of the World Natural Heritage as well as National Parks of Japan.

References

Hirano T, Kameda Y, Kimura K, Chiba S (2014) Substantial incongruence among the morphology, taxonomy, and molecular phylogeny of the land snails *Aegista*, *Landouria*, *Trishoplita*, and *Pseudobuliminus* (Pulmonata: Bradybaenidae) occurring in East Asia. Mol. Phylogenet Evol 70:171-181. https://doi.org/10.1016/j.ympev.2013.09.020

Okinawa Prefecture (2017) Threatened wildlife of Okinawa Prefecture: Red Data Okinawa. Okinawa: Nature Conservation Division, Department of Cultural and Environmental Affairs. 467-613. Revised edn (Animals) [in Japanese] https://www.pref.okinawa.jp/site/kankyo/shizen/hogo/okinawa_rdb_doubutu.html

Osozawa S, Oba Y, Kwon HY, Wakabayashi J (2015) Vicariance of *Pyrocoelia* fireflies (Coleoptera: Lampyridae) in the Ryukyu islands, Japan. Biol J Linn Soci 116(2):412-422. https://doi.org/10.1111/bij.12595
